# Metabolites from Microbes Isolated from the Skin of the Panamanian Rocket Frog *Colostethus panamansis* (Anura: Dendrobatidae)

**DOI:** 10.3390/metabo10100406

**Published:** 2020-10-13

**Authors:** Christian Martin H., Roberto Ibáñez, Louis-Félix Nothias, Andrés Mauricio Caraballo-Rodríguez, Pieter C. Dorrestein, Marcelino Gutiérrez

**Affiliations:** 1 Centro de Biodiversidad y Descubrimiento de Drogas, Instituto de Investigaciones Científicas y Servicios de Alta Tecnología (INDICASAT AIP), Clayton, Panama 0843-01103, Panama; cmartin@indicasat.org.pa; 2 Department of Biotechnology, Acharya Nagarjuna University, Nagarjuna Nagar, Guntur 522510, India; 3 Smithsonian Tropical Research Institute, Balboa, Ancon, Panama 0843-03092, Panama; ibanezr@si.edu; 4 Collaborative Mass Spectrometry Innovation Center, Skaggs School of Pharmacy and Pharmaceutical Sciences, University of California San Diego, La Jolla, CA 92093, USA; nothias@ucsd.edu (L.-F.N.); amcaraballor@ucsd.edu (A.M.C.-R.); pdorrestein@health.ucsd.edu (P.C.D.)

**Keywords:** 3D molecular cartography, *Colostethus panamansis*, Dendrobatidae, skin-associated bacteria, 16S rRNA sequencing, feature-based molecular networking

## Abstract

The Panamanian rocket frog *Colostethus panamansis* (family Dendrobatidae) has been affected by chytridiomycosis, a deadly disease caused by the fungus *Batrachochytrium dendrobatidis* (*Bd*). While there are still uninfected frogs, we set out to isolate microbes from anatomically distinct regions in an effort to create a cultivable resource within Panama for potential drug/agricultural/ecological applications that perhaps could also be used as part of a strategy to protect frogs from infections. To understand if there are specific anatomies that should be explored in future applications of this resource, we mapped skin-associated bacteria of *C. panamansis* and their metabolite production potential by mass spectrometry on a 3D model. Our results indicate that five bacterial families (Enterobacteriaceae, Comamonadaceae, Aeromonadaceae, Staphylococcaceae and Pseudomonadaceae) dominate the cultivable microbes from the skin of *C. panamansis*. The combination of microbial classification and molecular analysis in relation to the anti-*Bd* inhibitory databases reveals the resource has future potential for amphibian conservation.

## 1. Introduction

Among the three existing orders of amphibians, Caudata (salamanders), Gymnophiona (caecilians) and Anura (frogs and toads) [1], the last one is the most diverse [2] and also widely affected by the lethal amphibian disease chytridiomycosis, caused by the fungus *Batrachochytrium dendrobatidis* (*Bd*). *Bd* is responsible for the high rate of amphibian loss in the world, particularly in the Neotropics [3,4,5,6,7,8]. This pathogen mainly infects amphibians at the ventral coronal plane (abdomen, pelvic patch, pes and toes) [9]. However, not all amphibians that have been exposed to *Bd* in nature have been affected equally. Some of them have shown asymptomatic responses and, therefore, are considered to be resistant or tolerant [9,10].

*Colostethus panamansis* (family Dendrobatidae) is considered a least-concerned species regarding its conservation status, despite a decreasing population trend [11]. In fact, population declines of this species have not been noticed in the lowlands of Panama. In contrast, highland populations of *C. panamansis* in Panama have experienced dramatic declines [12]. Infection experiments have shown that *C. panamansis* is a susceptible species to *Bd*, causing death in infected frogs [13]. It was hypothesized that healthy frogs of this species could harbor bacterial symbionts on their skin as potential producers of specialized metabolites that could protect them against microbial pathogens [14,15,16,17,18,19]. While a small number of specialized metabolites produced by amphibian skin-associated bacteria has been reported, including some endowed with antifungal activity against *Bd* in vitro [17,20,21,22], multi-omics methods are needed to investigate the symbiotic metabolome of bacteria on the amphibian’s skin mucosome [23].

Recently, multi-omics approaches such as DNA sequencing and mass spectrometry-based metabolomics have allowed researchers to deeply explore the microbial and chemical diversity associated with a wide range of biological sources [24,25,26]. In the case of chemical diversity, the Global Natural Products Social molecular networking web-platform (GNPS) facilitates the exploration of the chemical space of metabolomes. GNPS is a tandem mass spectrometry (MS/MS) automated data organizational tool that makes comparisons of MS/MS fragmentation patterns among samples in order to cluster and visualize related molecules in a spectral network [27,28]. Feature-based molecular networking (FBMN) is a recent analysis method in GNPS that enhances quantitative capability and isomer resolution in molecular networks by coupling with mass spectrometry data processing tools, such as MZmine [29,30]. In addition, this makes it possible to annotate the spectra with computational tools, like SIRIUS [31] which can provide putative molecular formula and structural information. Researchers can perform three dimensional (3D) cartographic maps to explore the spatial distribution of host-derived molecules, along with bacterial specialized metabolites [32,33,34,35,36,37,38]. Characterization of skin microorganisms and their related molecules is essential for understanding host–microbial symbiont interactions. Although many studies have focused on the human-associated microbes, i.e., gut and skin, far less is known about the skin microbes of other mammals, amphibians, birds, fish, and reptiles [39].

In this study, we performed 3D molecular cartography of the bacterial genomics and metabolomics data obtained from bacterial isolates collected from different body locations of *C. panamansis* skin. The mapping results allowed us to correlate the distribution of the most abundant bacterial isolates with some of the specialized metabolites on multiple body parts of the frog, helping to understand the function of skin-associated bacteria in *C. panamansis*. Additionally, we detected non-described molecules in *C. panamansis* that could be produced by previously reported anti-*Bd* bacterial isolates. That means that such molecules could serve as potential sources for future conservation treatments in amphibian populations.

## 2. Results

### 2.1. 16S rRNA Amplicon Sequencing and Molecular Cartography

**Figure 1:**
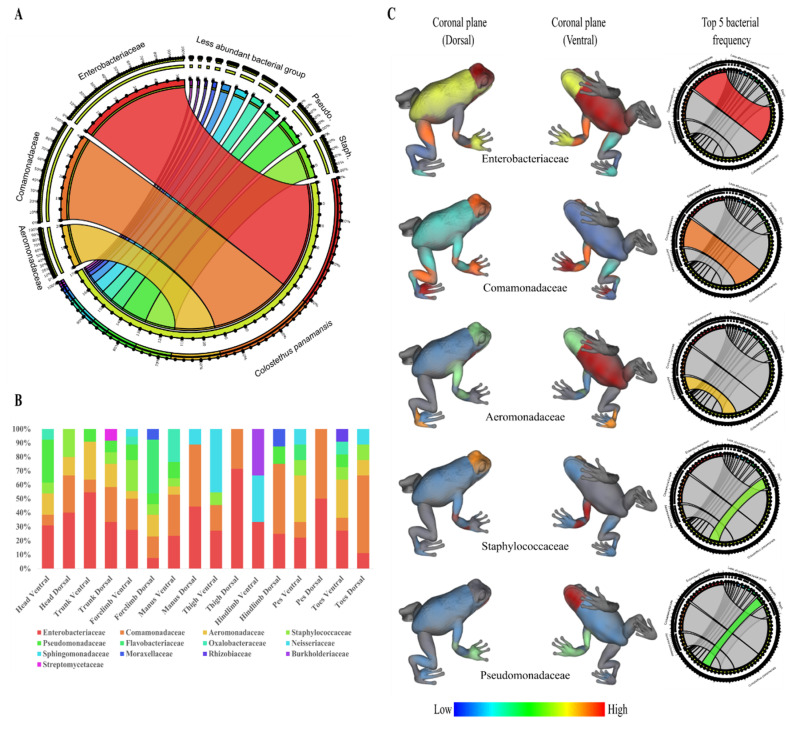
Frequency of isolated bacteria from the skin surface of *C. panamansis*. (**A**) Relative abundances of bacterial families from the skin of *C. panamansis* based on 16S rRNA amplicon sequencing. (**B**) The proportional abundance (as a percent of total sequences) of bacterial families based on body parts. (**C**) 3D topographic map of *C. panamansis* based on the five most abundant bacterial families isolated at each body location. Red represents the highest percentage of each family that was isolated and blue the lowest percentage.

We obtained 170 isolates from the skin of four specimens of *Bd*-uninfected frogs of *C. panamansis* collected in June 2016 and August 2017. Frogs collected in 2016 were tested for *Bd* infection through q-PCR, displaying negative results. After performing Sanger amplicon sequencing, we found that bacterial isolates belong to phylum Proteobacteria, Firmicutes, Bacteroidetes and Actinobacteria with a relative abundance of 85.3%, 10.6%, 3.5% and 0.6%, respectively. Within the phylum Proteobacteria, the largest group, the most frequent families were Enterobacteriaceae (31.2%), Comamonadaceae (24.1%), Aeromonadaceae (11.8%) and Pseudomonadaceae (7.7%). Within Bacteroidetes and Firmicutes, the bacterial families were mainly composed of Staphylococcaceae (8.8%) and Flavobacteriaceae (3.5%), respectively. Actinobacteria was composed by Streptomycetaceae (0.6%), being the less abundant phylum (Figure 1A). We also found significant differences between the number of isolates, based on bacterial families, and body parts sampled (dorsal and ventral: head, trunk, forelimbs, manus, thigh, hind limbs, pes and toes) (Friedman test; *p* = 0.0445). Significant differences between the number of bacterial isolates per family and the coronal plane (dorsal and ventral) of specimens collected (Pearson Chi-Square; *p* = 0.0096) were found.

In terms of topographical distribution, we showed the results of the five most frequent bacterial families of isolates from the skin of *C. panamansis*. We observed that: (1) Enterobacteriaceae was isolated mainly on the dorsal head, thighs, trunk and manus, and the ventral trunk, forelimbs, head, manus and thighs; (2) Comamonadaceae was isolated on the dorsal head/manus/hind limbs/toes and the ventral manus/forelimbs/thighs; (3) Aeromonadaceae was isolated on the dorsal head/pes/forelimbs and the ventral trunk/pes/toes/head; (4) Staphylococcaceae was isolated on the dorsal head and ventral forelimbs; and (5) Pseudomonadaceae was found on the dorsal manus and ventral head/forelimbs/manus (Figure 1B,C).

### 2.2. Molecular Networking and Molecular Cartography

**Figure 2:**
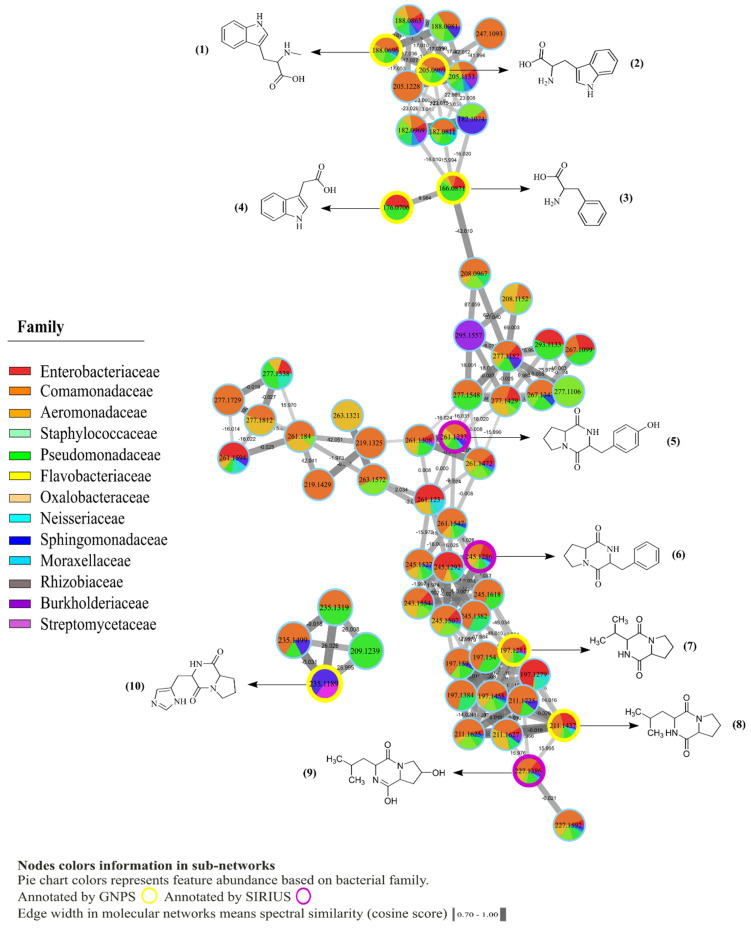
Molecular network of specialized metabolites (<300 Daltons) annotated in crude extracts of bacterial isolates from skin of *C. panamansis* by LC-MS/MS analysis. Highlighted nodes correspond to annotated metabolites by GNPS (yellow) and SIRIUS/CSIFingerID (purple). Pie charts inside nodes denotes abundance while colors correspond to the bacterial family where the feature was found. Edge width between nodes displays cosine score values, which are related to the spectral similarities between nodes.

**Figure 3:**
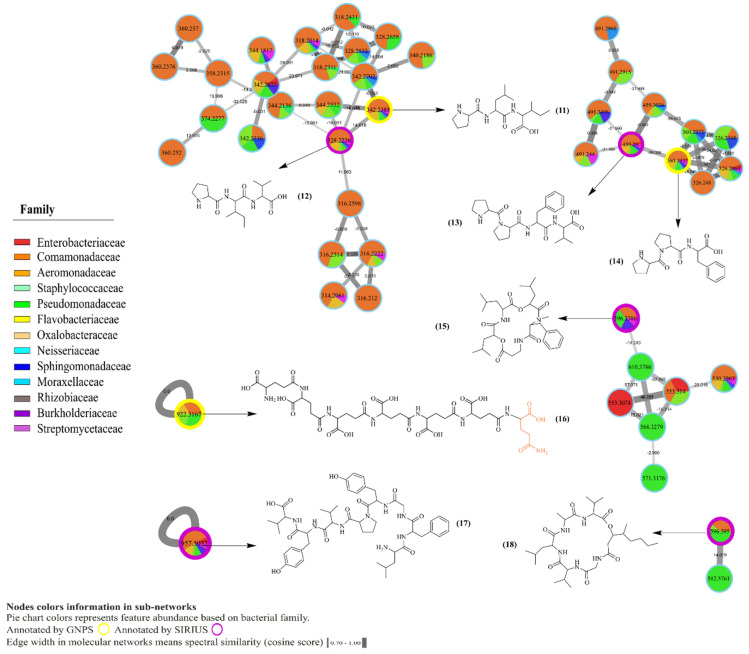
Molecular network of annotated peptides identified in crude extracts of bacterial isolates from skin of *C. panamansis* by LC-MS/MS analysis. Highlighted nodes correspond to annotated metabolites by GNPS (yellow) and SIRIUS CSIFingerID (purple). Pie charts inside nodes denotes abundance while colors correspond to the bacterial family where the feature was found. Edge width between nodes displays cosine score values, which are related to the spectral similarities between nodes.

MS/MS molecular networking of the 170 bacterial strains, 32 skin swabs and 2 pure compounds (tetrodotoxin and viscosin) revealed 2498 nodes. Each node represents an LC-MS/MS molecular feature that comprises detected *m*/*z*, MS/MS, and retention time. Thus, connected nodes generate clusters based on their spectral similarity in the molecular network. We focused on sub-networks containing GNPS and SIRIUS hits. The identified metabolites were detected from bacterial isolate extracts only. Putative annotations of compound and molecular families based on GNPS and SIRIUS matches correspond to level 2, respectively, according to Sumner et al. 2007 [40]. Eighteen selected bacterial metabolites, within *m*/*z* error of 10 ppm, were annotated as *N*-methyl tryptophan, *m*/*z* 205.0969 [M + H − H_2_O]^+^ (1), tryptophan, *m*/*z* 188.0696 [M + H − H_2_O]^+^ (2), phenylalanine, *m*/*z* 166.0871 [M + H]^+^ (3), 3-indole acetic acid, *m*/*z* 176.0706 [M + H]^+^ (4) and diketopiperazines, *m*/*z* 261.1233 [M + H]^+^ (5), *m*/*z* 245.1286 [M + H − H_2_O]^+^ (6), *m*/*z* 197.1281 [M + H]^+^ (7), *m*/*z* 211.1437 [M + H]^+^ (8), *m*/*z* 227.1386 [M + H]^+^ (9) and *m*/*z* 235.1189 [M + H − H_2_O]^+^ (10) (Figure 2), as well as the oligopeptides that included Pro-Leu-Ile, *m*/*z* 342.2383 [M + H]^+^ (11), Pro-Ile-Val, *m*/*z* 328.2226 [M + H]^+^ (12), Pro-Pro-Phe-Val, *m*/*z* 459.2600 [M + H]^+^ (13), Pro-Pro-Phe, *m*/*z* 360.1937 [M + H]^+^ (14), cyclodepsipeptide Leualacin, *m*/*z* 596.3386 [M + Na]^+^ (15), a poly-glutamic acid analog, *m*/*z* 922.3167 [M + H]^+^ (16), Leu-Phe-Gly-Tyr-Pro-Val-Tyr-Val, *m*/*z* 957.5032 [M + H]^+^ (17), and cyclo Ala-Val-3-hydroxy-4-methyloctanoyl-Gly-Val-Leu, *m*/*z* 596.3950 [M + H]^+^ (18) (Figure 3). After feature-based molecular networking analysis, both tetrodotoxin (TTX) and viscosin were absent in the bacterial crudes or swabs from the sampled specimens of *C. panamansis*.

**Figure 4:**
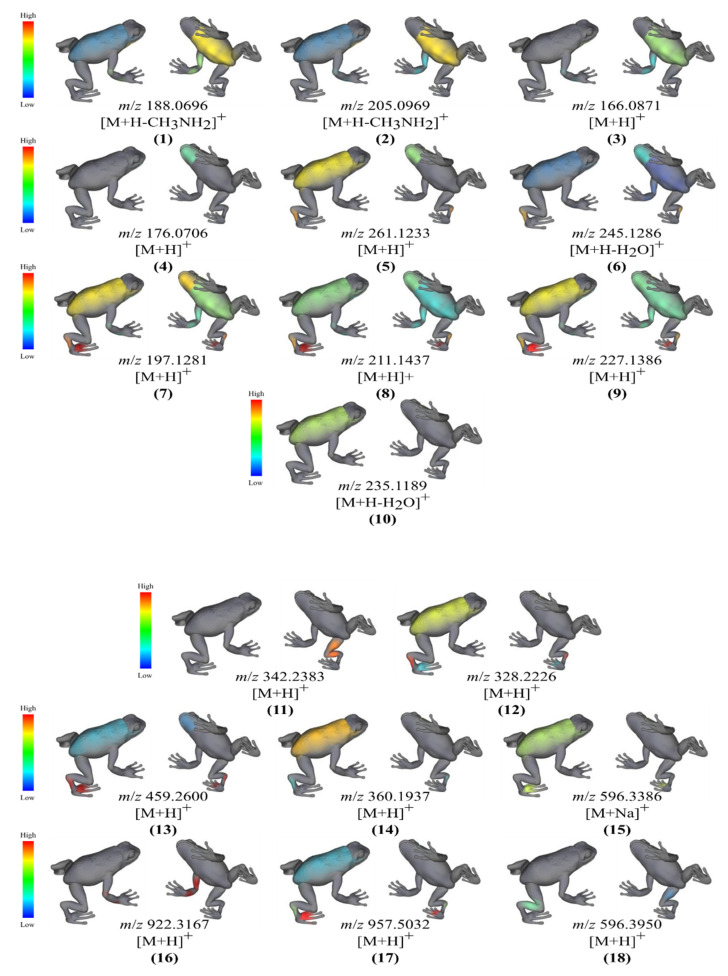
Molecular cartography of annotated metabolites identified from bacterial isolates sampled from the skin surface of *C. panamansis*. These metabolites were annotated as tryptophan derivatives (1,2), phenylalanine (3), 3-indoleacetic acid (4), diketopiperazines (5–10) and peptides (11–18). Intensities are related to the abundance of the specialized metabolites in the samples at each body part. Red represents the highest abundance while blue represents the lowest abundance.

Through integration of MS/MS data to ′ili web platform [33], we describe the molecular distribution of annotated specialized metabolites. Compounds 1, 2 and 3 were found on the ventral trunk samples. Compound 4 was found on the ventral head. Diketopiperazines-related metabolites were identified on multiple body parts. MS/MS features of compound 5 were found on the dorsal trunk/pes and ventral head and (6) on the dorsal pes and ventral head. Compounds 7, 8 and 9 were spotted at the dorsal toes, dorsal pes, dorsal trunk, ventral head, ventral trunk, and ventral forelimbs. MS/MS feature 10 was found in samples from the dorsal trunk (Figure 4).

We also visualized; annotated oligopeptides produced by skin-associated bacterial isolates through the ′ili web platform. The tripeptide 11 was found in samples from the ventral thigh and hind limbs while 12 was distributed among the dorsal pes, trunk, and toes. Compound 13 was observed in the dorsal pes, toes, trunk, and ventral head and 14 was found in samples from the dorsal trunk and pes. Compound 15 and 17 were both identified in dorsal trunk and toes samples. Compound 16 was associated with samples from forelimbs only. Compound 18 was related to samples from dorsal hind limbs and the ventral thigh (Figure 4).

**Figure 5:**
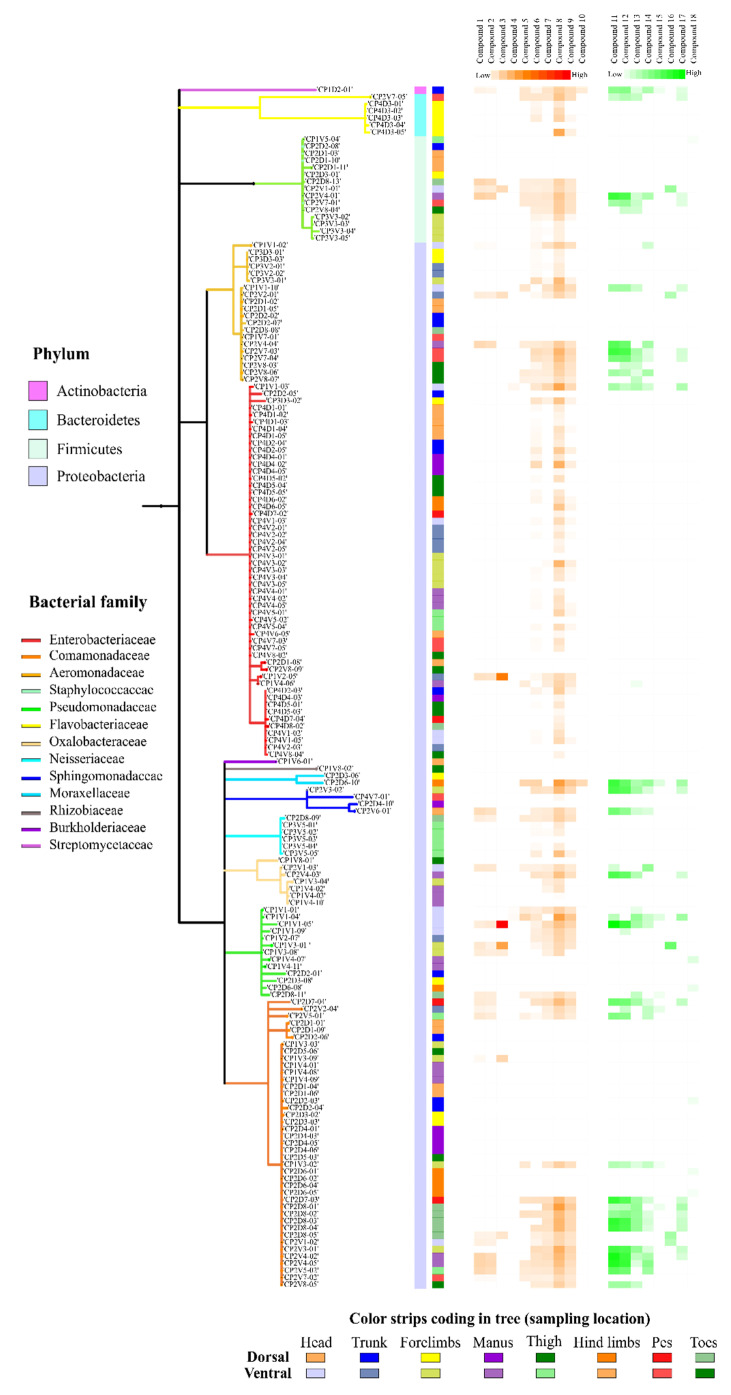
Phylogenetic tree and structures of annotated metabolites from 170 cultivable bacteria isolated from the skin of *C. panamansis* and a heatmap that displays the abundance of specialized metabolites annotated as molecular features by feature based molecular networking-analysis.

The specialized metabolites annotated through GNPS and SIRIUS were found in multiple samples (crude extracts from bacterial isolates) obtained from different body parts on the skin of *C. panamansis*. We found significant differences (Friedman test; *p* < 0.001) between bacterial families of isolates and the number of small molecules annotated. These small molecules were produced by bacterial isolates that belong to the families Enterobacteriaceae, Comamonadaceae, Pseudomonadaceae, Aeromonadaceae and Staphylococcaceae (Supplementary Materials Table S1, Figure 5). We also found significant differences (Friedman test; *p* < 0.001) when considering bacterial families and the number of peptides annotated. Such peptides were detected in well-defined bacterial families, namely, Comamonadaceae, Aeromonadaceae, Pseudomonadaceae and Staphylococcaceae (Table S2, Figure 5).

### 2.3. Comparison between 16S rRNA Sequences from C. panamansis and Bd-Inhibitory Public Datasets

**Figure 6:**
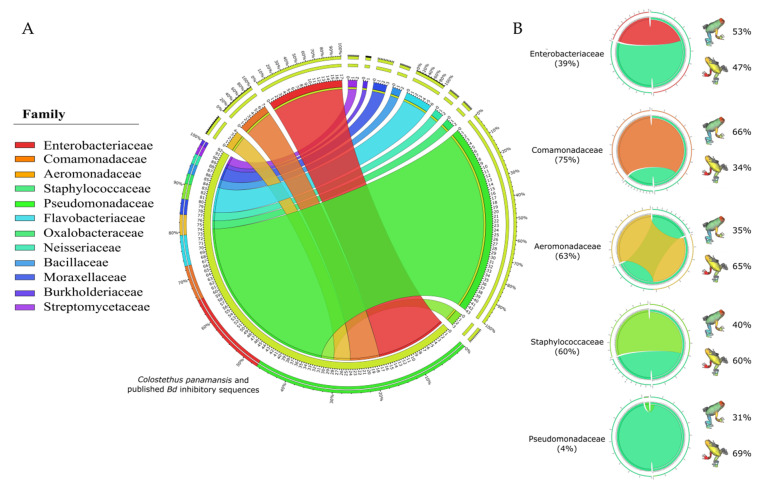
Relative abundances of 791 bacterial sequences from *C. panamansis* and anti-*Bd* published sequences. (**A**) Relative abundance of 16S rRNA sequences from *C. panamansis* and anti-*Bd* sequences from published databases based on their bacterial family. (**B**) Comparison between the five most abundant bacterial families in *C. panamansis* and *Bd*-inhibitory sequences (light green) and the distribution of isolates based on the coronal plane in *C. panamansis* is also displayed.

After comparing 170 bacterial sequences from isolates obtained from the skin of *C. panamansis* along with 621 bacterial sequences that displayed inhibitory effects against *Bd*, deposited in a public database [41], Pseudomonadaceae was the most abundant family (43%), followed by Enterobacteriaceae (17%), Comamonadaceae (7%), Aeromonadaceae (4%) and Staphylococcaceae (3%) (Figure 6A). We calculated the proportion of *C. panamansis* sequences within them as Enterobacteriaceae (39%), Comamonadaceae (75%), Aeromonadaceae (63%), Staphylococcaceae (60%) and Pseudomonadaceae (4%). Based on the coronal plane of isolates from *C. panamansis*, we found the major percentage samples from dorsal body parts in Enterobacteriaceae and Comamonadaceae (53% and 66%, respectively), while Aeromonadaceae, Staphylococcaceae and Pseudomonadaceae displayed on ventral body parts (65%, 60% and 69%, respectively) (Figure 6B).

## 3. Discussion

The primary role of animal skin is to serve as a physical barrier to protect the body against adverse effects from the environment and harmful organisms. Animal skin harbors a wide range of microbes due to direct contact with the environment. These microbes may produce specialized metabolites that could protect their host against pathogens [42]. In the case of amphibians, microbes are acquired from water, soil, plants and even other amphibians [18,43,44,45,46,47]. As *C. panamansis* is found in riparian habitats, a few meters from the water’s edge and under rocks, this may explain why the skin-associated bacteria in our samples mainly belong to the phylum Proteobacteria [46,48]. The high prevalence of this phylum was observed in other studies where culture-dependent and culture-independent approaches for bacterial identification were applied [22,46,49,50,51]. In terms of bacterial families, specimens of *C. panamansis* from the General de División Omar Torrijos Herrera National Park, in central Panama, mainly harbored cultivable bacteria of the families Enterobacteriaceae, Comamonadaceae, Aeromonadaceae, Staphylococcaceae and Pseudomonadaceae. However, the proportion of these bacterial families contrasted to previous reports in *C. panamansis* and other Panamanian frogs, where Pseudomonadaceae (*Pseudomonas* sp.) and Aeromonadaceae (*Aeromonas* sp.) were the most abundant bacterial families [22,46,49,50,51]. Although few studies have been carried out in tropical regions, such differences could be attributed to distinct genomic approaches applied (culture-dependent vs. culture independent), sampling sites, sampling size, host specimens and even host-susceptibility to *Bd* [22,46,52].

Through high-resolution mass spectrometry analyses, we obtained relevant information about specialized metabolites through molecular networking. This allows for the arrangement of large sets of tandem MS/MS data based on fragmentation pattern similarity, allowing the detection of molecules with related structures [27,28,53]. At present, the chemistry of specialized microbial metabolites on amphibian skin in tropical frogs and their possible protective and functional roles has not been studied in depth [22,49,50]. Considering that natural products and their derivatives constitute around half of the pharmaceuticals on the market today and provide for many essential agricultural products [54], eighteen specialized metabolites were annotated by means of GNPS and SIRIUS [27,31] and are reported here for the first time as metabolites produced by frog-associated bacteria.

Tryptophan derivatives such as 1 and 2, are well known for exhibiting antioxidant and immunomodulatory properties [55,56]. In terms of immunoregulatory properties, these skin-associated bacterial metabolites may influence the response from the host’s immune system to help fight off infection [57]. However, it is important to mention that *Bd* also produces tryptophan derivative molecules, which inhibit host immunity and even enhance the survival of this frog-killing fungus [58].

Phenylalanine 3 is an essential amino acid, known to be directly taken up from water, which promotes larval growth in the salamander *Hynobius retardatus* [59]. Considering that microbes can be transmitted vertically through parental care to the embryos [60,61], if such vertical transmission occurs in *C. panamansis* during egg attendance and tadpole transport, phenylalanine bacterial producers might serve as growth promoters at the larval stages of this frog. The indole-3-acetic acid 4 is a common biosynthetic product of tryptophan in bacteria through different biosynthetic pathways, which is produced by a wide range of microbes [62]. It is very well known for contributing to plant growth [62,63,64]; however, its presence and role on amphibian skin has not been reported. The diketopiperazines (5–10) have been isolated from diverse microorganisms [65,66], and exhibit a wide range of biological properties such as antimicrobials, immunosuppressant and anticancer [65,67,68,69,70]. This stresses the need for understanding how these skin-associated bacteria, which produce myriad natural products, could be further used for amphibian conservation strategies.

The antimicrobial peptides (AMPs) are small molecules with a broad spectrum of inhibitory effects against bacteria, fungi, protozoa and viruses [71]. AMPs have been reported in amphibian skin secretions for conferring host defenses against pathogens and predators [5,16]. In some cases, peptides secreted by amphibian skin glands may not negatively affect bacterial growth, including bacteria with anti-*Bd* properties [72]. Amphibian skin-associated bacteria are also known to produce peptides with anti-*Bd* properties [22]. Nonetheless, peptides of bacterial origin had not been described frequently in frogs even less in the family Dendrobatidae. In this study, we found bacterial oligopeptides composed mainly by alpha amino acids. These oligopeptides were compounds 11, 12, 13 and 14. It is known that oligopeptides containing proline, as part of amino acid residues, are able to target intracellular membranes in microbes, leading to cell death by lysis [71]. Additionally, 15 is a cyclic depsipeptide, known as leualacin, which does not exhibit antimicrobial activity; however, it has been reported to act as a blood pressure regulator in mice [73], and could potentially have a similar role in frogs.

Through the dereplicator workflow launched in GNPS, it was possible to extend the identification of peptides and other natural products [74]. Thus, we identified the poly-glutamic acid analog 16. This compound is a polymer composed of D-glutamic acid residues that have skin protectant properties, improving skin moisture and elasticity, even more than collagen and hyaluronic acid [75]. However, further studies are needed to determine whether this structural modification could provide the same or higher skin protection in amphibians. Additionally, poly-glutamic acid is known to exhibit high antibiotic activity against Gram positive and Gram negative bacteria, i.e., *Listeria monocytogenes*, *Stenotrophomonas typhimurium*, *Staphylococcus aureus*, *Klebsiella pneumoniae* and *Escherichia coli* [76].

The oligopeptide 17, was annotated with SIRIUS coupled to CSI:FingerID that is able to propose molecular structure candidates [31]. However, in a few cases the biological roles of predicted metabolites are unknown. Finally, compound 18 is a cyclic depsipeptide that has been reported as presenting antifungal properties [77]. Since cyclic lipodepsipeptides have been recently reported by our group to have biological in vitro activity against *Bd* [22], we infer a similar property for this bacterial metabolite isolated from the skin of *C. panamansis*.

3D molecular cartography studies have been implemented to integrate and interpret molecular results in large studies (i.e., genomics and metabolomics) from human, host-parasites and plant-related samples [32,35,36,78]; however, such studies have not been conducted in amphibians. By generating a 3D molecular cartography model of an amphibian, i.e., the Panamanian rocket frog *C. panamansis*, it was possible to visualize and understand the body distribution of skin-associated molecules and their bacterial producers [32,35,79]. Although isolation of amphibian’s strains is common, understanding their body distribution and their role in the environment can be helpful in the selection of isolates for further novel secondary metabolite discovery. In this sense, the topographical distribution is particularly relevant because of the amphibian-killing fungus, *B. dendrobatidis*, which infects vascularized body parts such as the ventral abdomen, pelvic patch, pes and toes of frogs [9,80,81]. Therefore, bacterial distribution and their specialized metabolites could represent key factors in studies related to amphibian conservation since significant differences between the number of bacterial isolates per family on ventral and dorsal body parts were found. Our results provide insight into how bacterial isolates on the skin of *C. panamansis* could differ based on the coronal plane. Such differences could be attributed to the environmental contact effect (ventral region) and the heterogeneity of skin gland (dorsal region) density on the coronal planes [82].

Bacterial gene sequences from isolates obtained from different body parts in *C. panamansis* were compared along with published sequences with anti-*Bd* properties [41]. We determined that a large amount of anti-*Bd* bacteria predominantly belongs to the family Pseudomonadaceae [22] while the families Enterobacteriaceae, Comamonadaceae, Aeromonadaceae and Staphylococcaceae comprised around thirty percent. Within these five bacterial families, a genetic dispersion is shown, as has also been shown in other amphibian-related studies [41]. It is important to mention that although bacterial isolates from *C. panamansis* have displayed a level of relationship with bacteria with anti-*Bd* properties published by Woodhams et al. 2015 [41], it needs to be determined experimentally that bacteria found in *C. panamansis* exhibit antifungal activity against *Bd* and other pathogens. Given the range of biological activities reported previously for compounds 1–18, including antimicrobial, we could suggest that multiple isolates from *C. panamansis* very likely have the potential to fend off pathogens in the natural environment through some of the specialized metabolites presented in this study. Our results indicate that the bacterial strains reported here indeed have the capacity to produce bioactive molecules. These molecules may have potential applications in medicine and agriculture and are related to strains producing anti-*Bd* natural products and certainly represent a strain repository for future studies on drug discovery and frog ecology.

## 4. Materials and Methods

### 4.1. Sampling Specimens

In June 2016 and August 2017, adult specimens of *C. panamansis* (*n* = 4, 2 males and 2 females) were collected from the trail La Rana Dorada (N: 8.67143°, W: 80.59025°, elevation: 625 m) at the General de División Omar Torrijos Herrera National Park, north of El Copé, Province of Coclé, Panama. We did not seek to describe general differences between males and females, which would require a larger sample size of each sex. Frogs were individually rinsed with sterile water to remove transient bacteria from the skin [60]. After rinsing, 8 cotton swabs pre-moistened with distilled water, 50:50 ethanol/deionized water, were used to sample the cultivable skin-associated bacteria and specialized metabolites, respectively. We also sampled two specimens collected in 2016 for testing infection caused by *Bd*, based on a quantitative PCR technique [83]. The sampling of body parts was performed 10 times on dorsal and ventral regions, as presented in the supplementary information. To determine *Bd* infection, only ventral regions at positions 2 and 5–8 were sampled (Figure S1) [84]. Swabs for cultivable bacteria were streaked on Petri dishes containing fresh R2A agar [85] (Becton, Dickinson and Company, Franklin Lakes, NJ, USA). Once bacterial colonies grew, they were characterized and codified according to morphological characteristics (i.e., form, color, texture, and border type). Isolates were streaked on R2A agar until pure colonies were obtained. The pure isolates were cryopreserved at −80 °C in R2A broth with 15% glycerol.

### 4.2. 16S rRNA Amplicon Sequencing of Cultivable Bacteria

Two universal primers, 27F (5′-AGAGTTTGATCCTGGCTCAG-3′) and 1492R (5′-GGTTACCTTGTTACGACTT-3′), were used in PCR to amplify the 16S rRNA gene to characterize the taxonomy of the cultured bacterial isolates. The amplification reactions were done in a total volume of 50 µL (45 µL of Master Mix and 5 µL of bacterial DNA). The reaction mixtures were amplified in a T3000 thermocycler (Biometra GmbH, Göttingen, Germany) at 95 °C for 5 min, followed by 30 cycles of 94 °C for 1 min, 55 °C for 1 min, 72 °C for 90 s, and a final elongation for 10 min. PCR products were verified by electrophoresis in 1% agarose gel. Amplicons were sent to Macrogen Inc. (Seoul, Korea) for Sanger sequencing. Furthermore, DNA sequences were cleaned and assembled using Geneious 8.1.9 (Biomatters, Auckland, New Zealand) [86]. The 16S taxonomic diversity was obtained by BLASTn. These results were uploaded to the web-platform iTOL (https://itol.embl.de/) and Circos (http://www.circos.ca/) for taxonomic visualization [87,88,89]. Gene sequences are available in GenBank with the accession code MK533936-MK534105.

### 4.3. Fermentation and Crude Extracts Preparation

Fresh bacterial colonies at 0.5 McFarland (1.00 × 10^6^ CFU/mL) were aseptically inoculated into 15 mL centrifuge tubes (Celltreat Scientific Products, Pepperell, MA, USA) containing 5 mL of fresh R2A broth. For fermentation, inoculated tubes were put in a MaxQ 3000 orbital shaker (Thermo Fisher Scientific, Waltham, MA, USA) at 225 rpm at room temperature for 10 days. Afterwards, the samples were centrifuged, and supernatants (3 mL) were collected for organic extraction with ethyl acetate (3 mL). The organic phase was retrieved and dried under vacuum in a Speed-Vac SC210A (Thermo Fisher Scientific, Waltham, MA, USA) for 24 h. Dry mass yield of the organic extracts is provided in Table S3.

### 4.4. LC-MS/MS Analysis

Organic extracts (0.05 mg) were reconstituted in LC-MS grade 80% MeOH/Water containing 2 µM sulfamethazine as internal standard. LC-MS/MS analysis was performed in an UltiMate 3000 UPLC system (Thermo Scientific) using a Scherzo SM-C18 (Imtakt, Portland, OR, USA) column (250 × 2 mm, 3 µm) and Maxis Q-TOF mass spectrometer (Bruker Daltonics, Fremont, CA, USA) equipped with an electrospray ionization source. Gradient elution was conducted with 100% solvent A (LC-MS grade 99.9% water, 0.1% formic acid) for 5 min, followed by a linear gradient from 100% A to 100% B (LC-MS grade 99.9% acetonitrile, 0.1% formic acid) in 5 min, held at 100% B for 2 min. Then, the process was repeated with 100% B to 100% A in 2.5 min and maintained at 100% A for 1 min, followed by a linear gradient from 100% A to 100% B in 2 min, held at 100% B for 1 min, then 100% B to 100% A in 1 min and held at 100% A for 1.5 min. A flow rate of 0.5 mL/min throughout the 21 min run was maintained. MS spectra were acquired in positive ion mode in the range of 100–2000 *m*/*z.* A mixture of 10 mg/mL of each sulfamethazine, sulfamethizole, sulfachloropyridazine, sulfadimethoxine, amitriptyline, and coumarin was run after every 96 injections for quality control. An external calibration with ESI-Low concentration tuning mix (Agilent technologies, Santa Clara, CA, USA) was performed prior to data collection and internal calibrant Hexakis (1H, 1H, 2H-perfluoro-ethoxy) phosphazene (CAS 186817-57-2) was used throughout the runs. The capillary voltage of 4500 V, nebulizer gas pressure (nitrogen) of 2 bar, ion source temperature of 200 °C, dry gas flow of 9 L/min source temperature, spectral rate of 3 Hz for MS1 and 10 Hz for MS2 was used. For acquiring MS/MS fragmentation, the 5 most intense ions per MS1 were selected [26]. The advanced stepping functions used to fragment ions and collision-induced dissociation (CID) energies for MS/MS data acquisition are presented in the supplementary information (Tables S4 and S5). The MS/MS active exclusion parameter was set to 2 and released after 30 s. The mass of internal calibrant was excluded from the MS/MS list using a mass range of *m*/*z* 621.5–623.0. The data was deposited in the MassIVE online repository (MSV000083487).

### 4.5. MS/MS Data Analysis and Molecular Networking

MS/MS data obtained from experiments were exported to 32-bit mzXML file, using Bruker Compass Data analysis v4.1. Feature-based molecular networking (FBMN) was performed (https://ccms-ucsd.github.io/GNPSDocumentation/featurebasedmolecularnetworking/), and these files were imported to MZmine 2.33 [29] for feature detection and then uploaded to the Global Natural Products Social Molecular Networking online platform (GNPS) [27]. Feature extraction for MS1 and MS2 was performed for a centroid mass detector with a signal threshold of 1.0 × 10^3^. Chromatogram builder was run with a minimum height of 1.0 × 10^3^ and tolerance of 20 ppm. Chromatograms were deconvoluted with a peak duration range of 0.0 to 1.00 min and a baseline cut-off algorithm of 1.0 × 10^3^. Isotopic peaks were grouped with a *m*/*z* tolerance of 0.02 Da and a retention time of 0.50 min. Detected peaks were aligned through the Join Aligner Module considering 0.02 Da and a retention time tolerance of 0.2 min. The molecular formula was also computed from isotope pattern analysis through in silico annotation using Sirius linked to the CSI:FingerID web service [31,90]. The MGF file generated from MZmine 2.33 was uploaded to GNPS for generating molecular networks of MS/MS spectra. Then, a molecular network was generated by filtering edges to have a cosine score above 0.70 and more than 4 matched peaks. The spectra in the network were then searched against GNPS public spectral libraries. The network and parameters can be accessed at the following link (https://gnps.ucsd.edu/ProteoSAFe/status.jsp?task=8e9ce0e180464ef69dff302dde800d3e). The dereplicator workflow and parameter can be accessed at https://gnps.ucsd.edu/ProteoSAFe/result.jsp?task=569e5436c7e34b208653f75e54c28079&view=view_significant_unique. This network was consequently imported to Cytoscape version 3.5.0 (www.cytoscape.org) for analysis.

### 4.6. 3D Modeling and Data Picturing

The 3D model of the frog was freely downloaded in STL format from https://www.cgtrader.com/free-3d-models/animals/other/stylised-frog-model. Coordinates (*x*, *y*, *z*) were manually created with MeshLab, labeled and exported into a CSV file [33]. Into this file, was inserted the bacterial diversity on the skin of *C. panamansis* obtained through Geneious 8.1.7 (Biomatters, Auckland, New Zealand) and the molecular features obtained from MZmine 2.33 GNPS. Data sets, genomics and metabolomics obtained from samples were merged, and the 3D model was generated through the ′ili online tool (https://ili.embl.de/) [33] by loading both files (.stl and .csv) described above. See supplementary information.

### 4.7. Bacterial Correlation for Inhibitory Properties Based on 16S rRNA Data

A total of 170 sequences from *C. panamansis* were analyzed along with 621 sequences from isolates that displayed in vitro inhibitory effects against *B. dendrobatidis* [41]. Inhibitory sequences (621) were selected based on their length (<1300 base pairs). All sequences were classified by using the Ribosomal Database Project plugin in Geneious R8.1.9 (Biomatters, Auckland, New Zealand). The resulting classification analysis is available at https://16s.geneious.com/16s/results/810efbc7-c031-40d7-b727-d88a2cb22cf2.html#. These results were uploaded to the web-platform Circos for visualization (http://www.circos.ca/) [87].

## 5. Conclusions

After integrating genomic and metabolomics data into a 3D molecular cartography, we were able to identify and determine the body distribution of compounds 1–18, which are reported for the first time as metabolites produced by frog cutaneous bacteria. We conclude that the most abundant bacterial families in specimens of the frog *C. panamansis* were Enterobacteriaceae, Comamonadaceae, Aeromonadaceae, Staphylococcaceae and Pseudomonadaceae. Isolates of such bacterial families were responsible for producing antimicrobial compounds such as methyl derivatives of tryptophan, derivatives of phenylalanine, diketopiperazines and peptides. Some of these molecules display a wide range of antimicrobial properties, which could lead future bioprospecting and amphibian conservation biology studies. We also found that both annotated molecules, small molecules and peptides, were widely distributed among bacterial families and are significantly different from anatomically coronal planes (dorsal and ventral) on the skin of *C. panamansis.* Such differences will highlight an alternative perspective for future isolation studies in amphibians and other host–microbial studies. Additionally, we determined that bacterial sequences from isolates obtained from different body parts on the skin of *C. panamansis* displayed a genetic dispersion among at least five bacterial families when compared with sequences of bacterial strains with *Bd*-inhibitory activity from public databases. This might suggest that multiple isolates from *C. panamansis*, potentially mediated by their specialized metabolites and their wide body distribution, could provide additional evidence of the chemical role of skin-associated bacteria in fending off fungal pathogens, such as *Bd*, in natural environments. Finally, viscosin, an anti-*Bd* peptide of bacterial origin, was not detected in this study and, although *C. panamansis* has been reported for presenting TTX, a water-soluble toxin on their skin; it was neither detected from bacterial crude extracts nor frog skin swabs.

## Supplementary Materials

The following are available online at https://www.mdpi.com/2218-1989/10/10/406/s1. Table S1. Frequency of small molecules based on their corresponding bacterial family producer. Table S2. Frequency of peptides based on their corresponding bacterial family producer. Table S3. Advanced stepping function used for ions fragmentation. Table S4. Collision-induced dissociation (CID) energies for MS/MS data acquisition. Table S5. Dry mass yield of the organic extracts. Figure S1. *Colostethus panamansis* sampling sites for MS/MS, cultivable bacteria identification and *Bd* infection analysis. Dorsal (L) and ventral (R). (1) Head, (2) trunk, (3) forelimb, (4) manus, (5) thigh, (6) hind limb, (7) pes and (8) toes. 3D files are available at: https://github.com/cmartinhdz/3D-molecular-cartography-of-the-Panamanian-rocket-frog-Colostethus-panamansis-Dendrobatidae-. Supporting information about annotated molecules is available at: https://github.com/cmartinhdz/3D-molecular-cartography-of-the-Panamanian-rocket-frog-Colostethus-panamansis-Dendrobatidae-/blob/master/Supporting%20information_annotated%20molecules.xlsx.
