# Supplementary material for: Metabolites from Microbes Isolated from the Skin of the Panamanian Rocket Frog Colostethus panamansis (Anura: Dendrobatidae)
Source: Metabolites. 2020 Oct 13;10(10):406. doi: 10.3390/metabo10100406 (PMC7601193; doi:10.3390/metabo10100406)
Supplement: Supplementary file 1 [file metabolites-10-00406-s001.pdf]

metabolites

Supplementary information

Metabolites from Microbes Isolated from the skin of the Panamanian rocket frog *Colostethus panamansis* (Anura: Dendrobatidae).

Christian Martin H. [1,2], Roberto Ibáñez [3], Louis-Félix Nothias [4], Andrés Mauricio Caraballo-Rodríguez [4], Pieter C. Dorrestein [4], Marcelino Gutiérrez [1*].

[1] Centro de Biodiversidad y Descubrimiento de Drogas, Instituto de Investigaciones Científicas y Servicios de Alta Tecnología (INDICASAT AIP), Clayton, Panama City, Panama. christian.martin.hdz@gmail.com (C.M.); mgutierrez@indicasat.org.pa (M.G.).

[2] Department of Biotechnology, Acharya Nagarjuna University, Nagarjuna Nagar, Guntur 522510, India. christian.martin.hdz@gmail.com (C.M.).

[3] Smithsonian Tropical Research Institute, Balboa, Ancón, Panama. ibanezr@si.edu (R.I.).

[4] Collaborative Mass Spectrometry Innovation Center, Skaggs School of Pharmacy and Pharmaceutical Sciences, University of California San Diego, California, USA. nothias@ucsd.edu (L-F. N.); amcaraballor@ucsd.edu (A.M.C.); pdorrestein@ucsd.edu (P.D.).

* Correspondence: mgutierrez@indicasat.org.pa; Tel.: +507-517-0732; Fax: +507-517-0701.

Table of contents.

- Table S1. Frequency of small molecules based on their corresponding bacterial family producer.
- Table S2. Frequency of peptides based on their corresponding bacterial family producer.
- Table S3. Dry mass yield of the organic extracts
- Table S4. Collision-induced dissociation (CID) energies for MS/MS data acquisition.
- Table S5. Advanced stepping function used for ions fragmentation.
- Figure S1. *Colostethus panamansis* sampling sites for MS/MS, cultivable bacteria identification and *Bd* infection analysis. Dorsal (L) and ventral (R). 1) Head, 2) trunk, 3) forelimb, 4) manus, 5) thigh, 6) hind limb, 7) pes and 8) toes.

- 3D files are available at: https://github.com/cmartinhdz/3D-molecular-cartography-of-the-Panamanian-rocket-frog-Colostethus-panamansis-Dendrobatidae-.
- Supporting information about annotated molecules available at: https://github.com/cmartinhdz/3D-molecular-cartography-of-the-Panamanian-rocket-frog-Colostethus-panamansis-Dendrobatidae-./blob/master/Supporting%20information_annotated%20molecules.xlsx

metabolites MDPI

Table S1. Frequency of detected molecular features from bacterial isolates based on their corresponding family.

	Small molecules									
Bacterial family	*m/z* 188.0696 (N=25)	*m/z* 205.0969 (N=24)	*m/z* 166.0870 (N=8)	*m/z* 176.0705 (N=3)	*m/z* 261.1233 (N=24)	*m/z* 245.1286 (N=85)	*m/z* 197.1281 (N=58)	*m/z* 211.1437 (N=112)	*m/z* 227.1386 (N=70)	*m/z* 235.1189 (N=2)
**Aeromonadaceae**	3	3	1	0	4	13	11	14	10	0
**Burkholderiaceae**	1	1	0	0	0	1	1	2	1	0
**Comamonadaceae**	10	9	2	0	7	14	18	18	18	0
**Enterobacteriaceae**	1	1	1	1	1	28	6	44	19	0
**Flavobacteriaceae**	0	0	0	0	1	4	1	5	3	0
**Moraxellaceae**	0	0	0	0	1	1	0	1	1	1
**Neisseriaceae**	1	1	0	0	1	5	2	5	2	0
**Oxalobacteraceae**	1	1	0	0	1	1	3	4	1	0
**Pseudomonadaceae**	4	4	3	2	3	7	7	8	7	1
**Rhizobiaceae**	0	0	0	0	0	1	0	0	0	0
**Sphingomonadaceae**	0	0	0	0	0	0	1	1	1	0
**Staphylococcaceae**	3	3	1	0	4	9	7	9	6	0
**Streptomycetaceae**	1	1	0	0	1	1	1	1	1	0

Table S2. Frequency in the number of bacterial isolates in which were detected molecular features (peptides) based on their corresponding family.

	**Peptides**							
**Bacterial family**	***m/z*** **342.2383 (N=30)**	***m/z*** **328.2226 (N=32)**	***m/z*** **459.26 (N=27)**	***m/z*** **360.1937 (N=26)**	***m/z*** **596.3386 (N=7)**	***m/z*** **922.3167 (N=5)**	***m/z*** **957.5032 (N=20)**	***m/z*** **596.395 (N=6)**
**Aeromonadaceae**	5	6	6	5	0	1	3	1
**Burkholderiaceae**	1	1	1	1	0	0	0	0
**Comamonadaceae**	14	15	12	12	3	2	9	2
**Enterobacteriaceae**	1	1	2	1	0	0	1	0
**Flavobacteriaceae**	1	1	1	0	0	0	1	0
**Moraxellaceae**	1	1	1	1	1	0	1	0
**Neisseriaceae**	0	0	0	0	0	0	0	0
**Oxalobacteraceae**	1	0	0	1	0	0	0	0
**Pseudomonadaceae**	2	2	4	2	2	1	0	2
**Rhizobiaceae**	0	0	0	0	0	0	1	0
**Sphingomonadaceae**	1	1	1	1	0	0	1	0
**Staphylococcaceae**	2	3	3	1	0	1	2	1
**Streptomycetaceae**	1	1	1	1	1	0	1	0

Table S3. Dry mass yield of the organic extracts

Number	Sample code	Weight 0	Weight f	gr	mg
1	CP1D2-01	4.6792	4.6794	0.0002	0.20
2	CP1V1-01	4.6842	4.6847	0.0005	0.50
3	CP1V1-02	4.6609	4.6632	0.0023	2.30
4	CP1V1-03	4.6836	4.6839	0.0003	0.30
5	CP1V1-04	4.6871	4.689	0.0019	1.90
6	CP1V1-05	4.649	4.6491	1E-04	0.10
7	CP1V1-09	4.6984	4.6985	1E-04	0.10
8	CP1V1-10	4.7038	4.7043	0.0005	0.50
9	CP1V2-05	4.6893	4.6896	0.0003	0.30
10	CP1V2-07	4.6758	4.6762	0.0004	0.40
11	CP1V3-01	4.6892	4.6897	0.0005	0.50
12	CP1V3-02	4.6927	4.6931	0.0004	0.40
13	CP1V3-03	4.6512	4.652	0.0008	0.80
14	CP1V3-04	4.6633	4.6638	0.0005	0.50
15	CP1V3-08	4.6722	4.6728	0.0006	0.60
16	CP1V3-09	4.6621	4.6629	0.0008	0.80
17	CP1V4-01	4.6655	4.6663	0.0008	0.80
18	CP1V4-02	4.6444	4.6446	0.0002	0.20
19	CP1V4-03	4.7061	4.7062	1E-04	0.10
20	CP1V4-06	4.6468	4.6472	0.0004	0.40
21	CP1V4-07	4.7313	4.7318	0.0005	0.50

22	CP1V4-08	4.7501	4.7505	0.0004	0.40
23	CP1V4-09	4.7033	4.7037	0.0004	0.40
24	CP1V4-10	4.6921	4.6926	0.0005	0.50
25	CP1V4-11	4.6857	4.6858	0.0001	0.10
26	CP1V5-04	4.6912	4.6916	0.0004	0.40
27	CP1V6-01	4.6832	4.6836	0.0004	0.40
28	CP1V7-01	4.6549	4.6552	0.0003	0.30
29	CP1V8-01	4.6702	4.6708	0.0006	0.60
30	CP1V8-02	4.649	4.6493	0.0003	0.30
31	CP2D1-01	4.6904	4.6905	1E-04	0.10
32	CP2D1-02	4.7281	4.7285	0.0004	0.40
33	CP2D1-03	4.6302	4.6303	1E-04	0.10
34	CP2D1-04	4.6571	4.6573	0.0002	0.20
35	CP2D1-05	4.7035	4.704	0.0005	0.50
36	CP2D1-06	4.7045	4.7046	1E-04	0.10
37	CP2D1-08	4.684	4.6845	0.0005	0.50
38	CP2D1-09	4.6465	4.6468	0.0003	0.30
39	CP2D1-10	4.6755	4.6762	0.0007	0.70
40	CP2D1-11	4.6527	4.6531	0.0004	0.40
41	CP2D2-01	4.6719	4.6724	0.0005	0.50
42	CP2D2-03	4.6795	4.6796	1E-04	0.10
43	CP2D2-05	4.6513	4.6525	0.0012	1.20
44	CP2D2-06	4.6688	4.6694	0.0006	0.60
45	CP2D2-07	4.7082	4.7093	0.0011	1.10

46	CP2D2-08	4.7017	4.7027	0.001	1.00
47	CP2D3-01	4.6525	4.6526	1E-04	0.10
48	CP2D3-02	4.7222	4.7227	0.0005	0.50
49	CP2D3-03	4.69	4.6909	0.0009	0.90
50	CP2D3-06	4.6573	4.6574	1E-04	0.10
51	CP2D3-08	4.7035	4.704	0.0005	0.50
52	CP2D4-01	4.7282	4.7292	0.001	1.00
53	CP2D4-03	4.7045	4.7047	0.0002	0.20
54	CP2D4-05	4.6467	4.647	0.0003	0.30
55	CP2D4-06	4.6754	4.6759	0.0005	0.50
56	CP2D4-08	4.6719	4.6725	0.0006	0.60
57	CP2D4-09	4.6527	4.6529	0.0002	0.20
58	CP2D4-10	4.6795	4.6799	0.0004	0.40
59	CP2D5-03	4.6615	4.6617	0.0002	0.20
60	CP2D5-06	4.6608	4.6613	0.0005	0.50
61	CP2D6-01	4.6798	4.6803	0.0005	0.50
62	CP2D6-02	4.6778	4.6782	0.0004	0.40
63	CP2D6-04	4.7126	4.713	0.0004	0.40
64	CP2D6-05	4.7068	4.7072	0.0004	0.40
65	CP2D6-08	4.6699	4.6707	0.0008	0.80
66	CP2D6-10	4.6878	4.6884	0.0006	0.60
67	CP2D7-03	4.6746	4.6755	0.0009	0.90
68	CP2D7-04	4.691	4.692	0.001	1.00
69	CP2D8-01	4.6993	4.6995	0.0002	0.20

70	CP2D8-02	4.6827	4.6828	0.0001	0.10
71	CP2D8-03	4.7069	4.7072	0.0003	0.30
72	CP2D8-04	4.6968	4.697	0.0002	0.20
73	CP2D8-05	4.6592	4.6593	1E-04	0.10
74	CP2D8-08	4.7049	4.705	1E-04	0.10
75	CP2D8-09	4.703	4.7032	0.0002	0.20
76	CP2D8-11	4.6942	4.6947	0.0005	0.50
77	CP2D8-13	4.6809	4.6811	0.0002	0.20
78	CP2V1-01	4.6815	4.6821	0.0006	0.60
79	CP2V1-02	4.6764	4.6766	0.0002	0.20
80	CP2V1-03	4.6586	4.6592	0.0006	0.60
81	CP2V2-01	4.6762	4.6766	0.0004	0.40
82	CP2V2-04	4.6622	4.6625	0.0003	0.30
83	CP2V3-01	4.6958	4.6959	1E-04	0.10
84	CP2V3-02	4.7428	4.7432	0.0004	0.40
85	CP2V4-01	4.65	4.6505	0.0005	0.50
86	CP2V4-02	4.6828	4.6829	1E-04	0.10
87	CP2V4-03	4.6864	4.6865	1E-04	0.10
88	CP2V4-04	4.6668	4.6674	0.0006	0.60
89	CP2V4-05	4.6388	4.6391	0.0003	0.30
90	CP2V5-01	4.6777	4.678	0.0003	0.30
91	CP2V5-02	4.6511	4.6515	0.0004	0.40
92	CP2V6-01	4.7036	4.7038	0.0002	0.20
93	CP2V7-01	4.7204	4.7208	0.0004	0.40

94	CP2V7-02	4.6435	4.644	0.0005	0.50
95	CP2V7-03	4.7033	4.7037	0.0004	0.40
96	CP2V7-04	4.6956	4.6959	0.0003	0.30
97	CP2V7-05	4.7645	4.7649	0.0004	0.40
98	CP2V8-03	4.7306	4.7307	1E-04	0.10
99	CP2V8-04	4.7317	4.7321	0.0004	0.40
100	CP2V8-05	4.746	4.7466	0.0006	0.60
101	CP2V8-06	4.724	4.7245	0.0005	0.50
102	CP2V8-07	4.7872	4.7878	0.0006	0.60
103	CP2V8-09	4.7441	4.7442	1E-04	0.10
104	CP3D3-01	4.7383	4.7386	0.0003	0.30
105	CP3D3-02	4.7736	4.7737	1E-04	0.10
106	CP3D3-03	4.639	4.6398	0.0008	0.80
107	CP3V2-01	4.7654	4.7656	0.0002	0.20
108	CP3V2-02	4.6806	4.6812	0.0006	0.60
109	CP3V3-01	4.6817	4.6822	0.0005	0.50
110	CP3V3-02	4.7068	4.7075	0.0007	0.70
111	CP3V3-03	4.664	4.6641	0.0001	0.10
112	CP3V3-04	4.6806	4.6808	0.0002	0.20
113	CP3V3-05	4.6808	4.6813	0.0005	0.50
114	CP3V5-01	4.7402	4.7407	0.0005	0.50
115	CP3V5-02	4.7358	4.7364	0.0006	0.60
116	CP3V5-03	4.7021	4.7025	0.0004	0.40
117	CP3V5-04	4.7159	4.7163	0.0004	0.40

118	CP3V5-05	4.6981	4.6991	0.001	1.00
119	CP4D1-01	4.697	4.6974	0.0004	0.40
120	CP4D1-02	4.7264	4.7265	1E-04	0.10
121	CP4D1-03	4.7192	4.7197	0.0005	0.50
122	CP4D1-04	4.716	4.7166	0.0006	0.60
123	CP4D1-05	4.7318	4.7324	0.0006	0.60
124	CP4D2-03	4.7371	4.7374	0.0003	0.30
125	CP4D2-04	4.7416	4.7419	0.0003	0.30
126	CP4D2-05	4.687	4.6873	0.0003	0.30
127	CP4D3-01	4.7397	4.7398	1E-04	0.10
128	CP4D3-02	4.7322	4.7326	0.0004	0.40
129	CP4D3-03	4.7161	4.7165	0.0004	0.40
130	CP4D3-04	4.704	4.7043	0.0003	0.30
131	CP4D3-05	4.7377	4.7382	0.0005	0.50
132	CP4D4-01	4.7791	4.7792	0.0001	0.10
133	CP4D4-02	4.7218	4.7222	0.0004	0.40
134	CP4D4-03	4.6666	4.6668	0.0002	0.20
135	CP4D4-05	4.7122	4.7124	0.0002	0.20
136	CP4D5-01	4.7302	4.7304	0.0002	0.20
137	CP4D5-02	4.7432	4.7433	1E-04	0.10
138	CP4D5-03	4.7591	4.7595	0.0004	0.40
139	CP4D5-04	4.7365	4.7366	1E-04	0.10
140	CP4D5-05	4.7278	4.7283	0.0005	0.50
141	CP4D6-02	4.7311	4.7314	0.0003	0.30

142	CP4D6-05	4.7477	4.748	0.0003	0.30
143	CP4D7-02	4.7266	4.7268	0.0002	0.20
144	CP4D7-04	4.7376	4.7382	0.0006	0.60
145	CP4D8-02	4.606	4.6065	0.0005	0.50
146	CP4V1-02	4.6375	4.6377	0.0002	0.20
147	CP4V1-03	4.6112	4.6114	0.0002	0.20
148	CP4V1-05	4.6252	4.6254	0.0002	0.20
149	CP4V2-01	4.622	4.6226	0.0006	0.60
150	CP4V2-02	4.5901	4.5906	0.0005	0.50
151	CP4V2-03	4.6302	4.6307	0.0005	0.50
152	CP4V2-04	4.6275	4.6278	0.0003	0.30
153	CP4V2-05	4.6332	4.6339	0.0007	0.70
154	CP4V3-01	4.6302	4.6306	0.0004	0.40
155	CP4V3-02	4.6196	4.6198	0.0002	0.20
156	CP4V3-03	4.6186	4.6191	0.0005	0.50
157	CP4V3-04	4.6316	4.632	0.0004	0.40
158	CP4V3-05	4.6021	4.6023	0.0002	0.20
159	CP4V4-01	4.6322	4.6325	0.0003	0.30
160	CP4V4-02	4.6277	4.6286	0.0009	0.90
161	CP4V4-05	4.6084	4.609	0.0006	0.60
162	CP4V5-01	4.6281	4.6287	0.0006	0.60
163	CP4V5-02	4.6124	4.6128	0.0004	0.40
164	CP4V5-04	4.6123	4.6127	0.0004	0.40
165	CP4V6-05	4.6032	4.6036	0.0004	0.40

166	CP4V7-01	4.6233	4.6238	0.0005	0.50
167	CP4V7-03	4.5994	4.6002	0.0008	0.80
168	CP4V7-05	4.6106	4.6109	0.0003	0.30
169	CP4V8-02	4.631	4.6314	0.0004	0.40
170	CP4V8-04	4.6062	4.6065	0.0003	0.30
171	CP1VENT01	4.6099	4.6102	0.0003	0.30
172	CP1VENT02	4.6064	4.6067	0.0003	0.30
173	CP1VENT03	4.5993	4.5995	0.0002	0.20
174	CP1VENT04	4.611	4.6112	0.0002	0.20
175	CP1VENT05	4.6178	4.6184	0.0006	0.60
176	CP1VENT06	4.6001	4.6002	1E-04	0.10
177	CP1VENT07	4.6264	4.6268	0.0004	0.40
178	CP1VENT08	4.6232	4.6233	0.0001	0.10
179	CP1DORS01	4.6207	4.6208	1E-04	0.10
180	CP1DORS02	4.6058	4.606	0.0002	0.20
181	CP1DORS03	4.6161	4.6162	1E-04	0.10
182	CP1DORS04	4.6063	4.6064	1E-04	0.10
183	CP1DORS05	4.612	4.6126	0.0006	0.60
184	CP1DORS06	4.6305	4.631	0.0005	0.50
185	CP1DORS07	4.6073	4.6075	0.0002	0.20
186	CP1DORS08	4.5989	4.599	1E-04	0.10
187	CP2VENT01	4.6085	4.6091	0.0006	0.60
188	CP2VENT02	4.6042	4.6047	0.0005	0.50
189	CP2VENT03	4.5912	4.5916	0.0004	0.40

190	CP2VENT04	4.6311	4.6317	0.0006	0.60
191	CP2VENT05	4.5816	4.5818	0.0002	0.20
192	CP2VENT06	4.5904	4.5905	1E-04	0.10
193	CP2VENT07	4.6082	4.6086	0.0004	0.40
194	CP2VENT08	4.6228	4.6233	0.0005	0.50
195	CP2DORS01	4.6024	4.6025	1E-04	0.10
196	CP2DORS02	4.5867	4.587	0.0003	0.30
197	CP2DORS03	4.6058	4.6062	0.0004	0.40
198	CP2DORS04	4.6068	4.607	0.0002	0.20
199	CP2DORS05	4.61	4.6103	0.0003	0.30
200	CP2DORS06	4.609	4.6095	0.0005	0.50
201	CP2DORS07	4.6243	4.6256	0.0013	1.30
202	CP2DORS08	4.6214	4.622	0.0006	0.60
203	CP3VENT01	4.6063	4.6067	0.0004	0.40
204	CP3VENT02	4.6262	4.6271	0.0009	0.90
205	CP3VENT03	4.6384	4.6392	0.0008	0.80
206	CP3VENT04	4.6146	4.6153	0.0007	0.70
207	CP3VENT05	4.5996	4.5997	0.0001	0.10
208	CP3VENT06	4.6566	4.6576	0.001	1.00
209	CP3VENT07	4.6101	4.6109	0.0008	0.80
210	CP3VENT08	4.6261	4.6268	0.0007	0.70
211	CP3DORS01	4.6358	4.6368	0.001	1.00
212	CP3DORS02	4.589	4.5893	0.0003	0.30
213	CP3DORS03	4.6274	4.628	0.0006	0.60

214	CP3DORS04	4.6185	4.6194	0.0009	0.90
215	CP3DORS05	4.6117	4.6122	0.0005	0.50
216	CP3DORS06	4.6456	4.6462	0.0006	0.60
217	CP3DORS07	4.6123	4.613	0.0007	0.70
218	CP3DORS08	4.6423	4.6429	0.0006	0.60
219	CP4VENT01	4.6243	4.6251	0.0008	0.80
220	CP4VENT02	4.5985	4.5987	0.0002	0.20
221	CP4VENT03	4.6283	4.6289	0.0006	0.60
222	CP4VENT04	4.6357	4.6362	0.0005	0.50
223	CP4VENT05	4.6146	4.6151	0.0005	0.50
224	CP4VENT06	4.6152	4.6155	0.0003	0.30
225	CP4VENT07	4.7386	4.739	0.0004	0.40
226	CP4VENT08	4.7145	4.715	0.0005	0.50
227	CP4DORS01	4.7162	4.7165	0.0003	0.30
228	CP4DORS02	4.728	4.7289	0.0009	0.90
229	CP4DORS03	4.7716	4.7719	0.0003	0.30
230	CP4DORS04	4.7535	4.7542	0.0007	0.70
231	CP4DORS05	4.7176	4.7185	0.0009	0.90
232	CP4DORS06	4.696	4.6969	0.0009	0.90
233	CP4DORS07	4.489	4.4893	0.0003	0.30
234	CP4DORS08	4.6996	4.6997	1E-04	0.10
235	CTRLR2A	4.6591	4.6595	0.0004	0.40
236	CTRLSWAB	4.4989	4.499	1E-04	0.10

metabolites

49 Table S4. Collision energies applied in a Tandem Time-of-Flight (TOF/TOF) Mass
50 Spectrometer for *C. panamansis* samples.

Type	Mass	Width	Collision	Charge State
Base	100.00	4.00	22.00	1
Base	100.00	4.00	18.00	2
Base	300.00	5.00	27.00	1
Base	300.00	5.00	22.00	2
Base	500.00	6.00	35.00	1
Base	500.00	6.00	30.00	2
Base	1000.00	8.00	45.00	1
Base	1000.00	8.00	35.00	2
Base	2000.00	10.00	50.00	1

Base	2000.00	10.00	50.00	2

Table S5. Collision ratio frequency, times and transfer time used during LC-MS/MS runs for samples taken from the skin of *C. panamansis*.

Time	Collision RF	Transfer Time	Collision
0	450.0	70.0	125
25	550.0	75.0	100
50	800.0	90.0	100
75	1100.0	95.0	75

Figure S1. Body parts sampled (dorsal (L) and ventral (R) regions) on the skin of *C. panamansis* for MS, cultivable bacteria and Bd infection analysis. Numbers are represented as follows: 1) Head, 2) trunk, 3) forelimb, 4) manus, 5) thigh, 6) hind limbs, 7) pes and 8) toes.

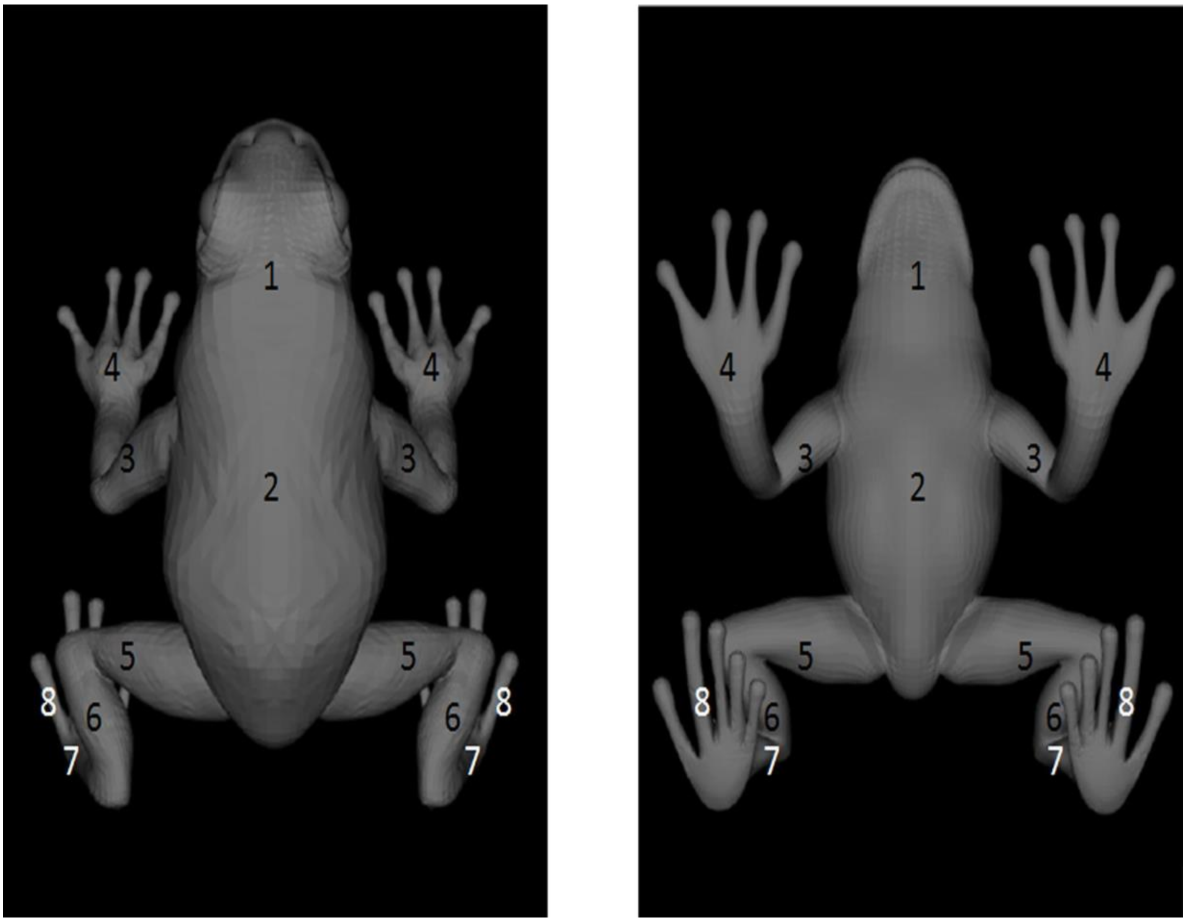

© 2019 by the authors. Submitted for possible open access publication under the terms and conditions of the Creative Commons Attribution (CC BY) license (http://creativecommons.org/licenses/by/4.0/).
